# A Study of MgZnO Thin Film for Hydrogen Sensing Application

**DOI:** 10.3390/ma17153677

**Published:** 2024-07-25

**Authors:** Tien-Chai Lin, Jyun-Yan Wu, Andres Joseph John Mendez, Nadir Salazar, Hao-Lin Hsu, Wen-Chang Huang

**Affiliations:** 1 Department of Electrical Engineering, Kun Shan University, No.195, Kunda Rd., Yongkang Dist., Tainan City 710303, Taiwan; tienchai@mail.ksu.edu.tw (T.-C.L.);; 2 Department of Mechanical Engineering, Kun Shan University, No.195, Kunda Rd., Yongkang Dist., Tainan City 710303, Taiwan; anderson.mendez769@gmail.com (A.J.J.M.); nadzsalazar1@gmail.com (N.S.); 3 Green Energy Technology Research Center, Kun Shan University, No.195, Kunda Rd., Yongkang Dist., Tainan City 710303, Taiwan; diago661228@stust.edu.tw

**Keywords:** MgZnO, hydrogen, gas sensor, thin film, RF co-sputtering

## Abstract

This research introduces a hydrogen sensor made from a thin film of magnesium zinc oxide (MgZnO) deposited using a technique called radiofrequency co-sputtering (RF co-sputtering). Separate magnesium oxide (MgO) and zinc oxide (ZnO) targets were used to deposit the MgZnO film, experimenting with different deposition times and power levels. The sensor performed best (reaching a sensing response of 2.46) when exposed to hydrogen at a concentration of 1000 parts per million (ppm). This peak performance occurred with a MgZnO film thickness of 432 nanometers (nm) at a temperature of 300 °C. Initially, the sensor’s responsiveness increased as the film thickness grew. This is because thicker films tend to have more oxygen vacancies, which are imperfections that play a role in the sensor’s function. However, further increases in film thickness beyond the optimal point harmed performance. This is attributed to the growth of grains within the film, which hindered its effectiveness. X-ray diffraction (XRD) and field-emission scanning electron microscopy (FE-SEM) were employed to thoroughly characterize the quality of the MgZnO thin film. These techniques provided valuable insights into the film’s crystal structure and morphology, crucial factors influencing its performance as a hydrogen sensor.

## 1. Introduction

Hydrogen shows some unusual properties such as a very low density (0.0899 kg/m^3^), high diffusion coefficient (0.61 cm^2^/s), and low boiling point (20.39 K). For the combustion characteristics of hydrogen, it shows a low minimum ignition energy of 0.017 mJ, high heat of combustion of 142 kJ/g, and wide flammable range from 4 to 75% [1,2]. Hydrogen also acts as a strong reducing agent for many elements and has a high permeability through many materials. All these facts stated above indicate the risks of hydrogen leakage. Therefore, an accurate hydrogen sensor is an essential device in the early warning system to prevent explosions during production, storage, transportation, and industrial applications [3,4,5].

Resistive-type transition metal oxide (TMO)-based thin film gas sensors have been widely used for the detection of toxic [6,7], explosive [8], and combustible gasses [9,10,11] for the safety of human beings, environmental monitoring, and air-quality control. They have drawn more attention because of their high sensitivity, simple structure, low-cost fabrication, long-term stability, and high potential to provide extremely small and low power-consuming sensors. The sensing mechanism of the metal oxide semiconductor resistive-type gas sensor is based on the variation of conductivity in the presence and absence of the test gas. Semiconducting metal oxides such as SnO_2_ [7,11,12], GaO [13], ZnO [14,15], NiO [14,16], In_2_O_3_ [17], and V_2_O_5_ [18] have been reported. These semiconducting metal oxides normally are polycrystalline and operate in the temperature range of 200~400 °C as they are applied to be a gas sensor. Their sensibility was evaluated through the change in conductivity in the application of a test gas. The performance of the polycrystalline semiconducting metal oxide sensor depends on the parameters including the crystalline phase [13], grain size [14], porosity, surface morphology [15,16], dopant type [17,18], etc.

ZnO is considered a promising candidate as a host material for gas sensing. The sensing characteristic of the ZnO-based sensor would effectively improve by doping certain elements [14,15]. Mg-doped ZnO materials exhibit several advantages of a controllable bandgap [19], less mismatch with ZnO, and good crystal quality with few oxygen vacancies [20,21]. There is some research related to the Mg-doped ZnO gas sensors. Liu et al. [22] proposed a pulsed laser deposition (PLD)-deposited MgZnO film and compared it to the undoped ZnO film as a hydrogen sensor. The sensor response is 2.9 for undoped ZnO film to 5000 ppm H_2_ at 300 °C. The gas response increased to about 50 for the MZO film measured under the same condition. The improved H_2_ sensing property of the MZO film might result from its smaller conductivity of it. Vijayalakshmi et al. [23] reported magnesium-doped zinc oxide (ZnO:Mg) thin films on ITO-coated glass substrates by the spray pyrolysis method. The introduction of Mg^2+^ into the ZnO matrix changed the grain shape and the grain size. The high reactivity efficiency to H_2_ for the Mg-dopant ZnO film is due to the high surface activity for smaller ZnO particles on the ITO lattice. K. Vijayalakshmi et al. [24] further discussed the Mg: ZnO nanorods by radio frequency sputtering for different substrate temperatures. The Mg: ZnO sensor prepared at 1200 °C revealed a fast response and recovery time of about 85 s and 70 s. Y. Adachi [25] reported enhanced H_2_ gas sensing properties of ZnO films by Mg alloying, and a sensor with a thickness of 9 nm, Mg_0.19_Zn_0.81_O, shows a maximum response value of 3000 to 800 ppm H_2_. The reason for the improved sensing properties is a reduced residual electron concentration in the films due to the substitution of Mg and the chemical sensitization effect of Mg atoms.

In this paper, we study a MgZnO thin film-based hydrogen sensor. The MgZnO thin film was prepared through an RF co-sputtering system. The contact electrode of the MgZnO sensor was deposited metal Pt by sputtering to achieve a hydrogen sensor device. The thickness effect of the MgZnO thin film and the measurement temperature effect of the sensor are stressed. 

## 2. Experiments

A P-type silicon substrate with a resistivity of 1–10 Ω-cm was firstly cleaned with a standard cleaning procedure: removing any surface contamination using acetone, methanol, and DI water in sequence for 5 min, respectively, and then drying with the dry nitrogen flow. The dimensions of 1.5 cm × 1.5 cm of a silicon chip were cut for the MgZnO deposition and processed to be a sensor. The MgZnO thin films were prepared by the RF magnetron co-sputtering equipped with a ZnO target (99.99% of purity, 7.62 cm (about 3 inches) in diameter) and MgO (99.99% of purity, 7.62 cm (about 3 inches) in diameter). The base pressure was 7 × 10^−5^ Torr and the working pressure was constant at 3 mTorr. Pure argon (99.99% purity) was used to be the sputtering gas. The RF powers toward the ZnO target and MgO target were 125 and 100 W, respectively. The deposition time of the MgZnO thin film varied from 10, 20, 40, to 80 min to obtain different thicknesses of the films. The structural, morphological, and optical characteristics of the MgZnO films were analyzed. Then, the electrode resistive-type hydrogen sensor was processed on the MgZnO thin film. The same sputtering system was used to deposit Pt electrodes with a thickness of about 100 nm on top of the MgZnO thin film. The DC power toward the Pt target was 60 W and the sputtering gas was argon with a flow rate of 4 sccm. The thickness of the MgZnO thin film was measured by a profilometer (Dektak 6M stylus profilometer, Veeco, Plainview, NY, USA) after various powers of deposition.

The crystallinity of MgZnO films was detected by using an X-ray diffractometer (XRD) on a Rigaku 18 KW Rotating Anode X-ray Generator with a continuous scan of Cu Kα radiation at λ = 1.54 Å. The surface morphology was observed by using a field-emission scanning electron microscope (Zeiss Supra 55 FE-SEM, Jena, Germany) with the acceleration voltage of 15 kV. The sensing characteristics of the device were evaluated through static and dynamic measurements. The relative humidity (RH) value is lower than 50% during the exposure. The size of the cylinder chamber is 40 cm in diameter with a height of 45 cm. The chamber was cleaned through a N_2_ purge before and after each exposure. The hydrogen concentration is controlled by the flux ratio between pure hydrogen and nitrogen gas. The response of the sensor to hydrogen gas was measured in a gas-controlled environment. The hydrogen sensor was placed in a quartz chamber. The probes that contact the inter-digital electrodes were connected to a General-Purpose Source meter (Keithley 2400). The temperatures of measurement varied at room temperature and 200 and 300 °C, respectively. The resistance measured in a gas environment was designated as *R_a_*, and that in an environment mixed with hydrogen and air was designated as *R_g_*. The sensing response of the measurement is defined as $Response=R_{a}/R_{g}$.

## 3. Results and Discussion

### 3.1. Thickness Effect of MgZnO Thin Film

The MgZnO thin film was deposited through a co-sputtering system. Both the ZnO and MgO ceramic targets were used to be the deposition source and their corresponding power is 125 and 100 W, respectively. The deposition time of the films varied from 10, 20, 40, to 80 min, respectively. The thickness of the film was achieved by an α-stepper and the corresponding thickness of each film is 132, 224, 423, and 836 nm, respectively. The thickness of the film is linearly dependent on the time of deposition. 

**Figure 1:**
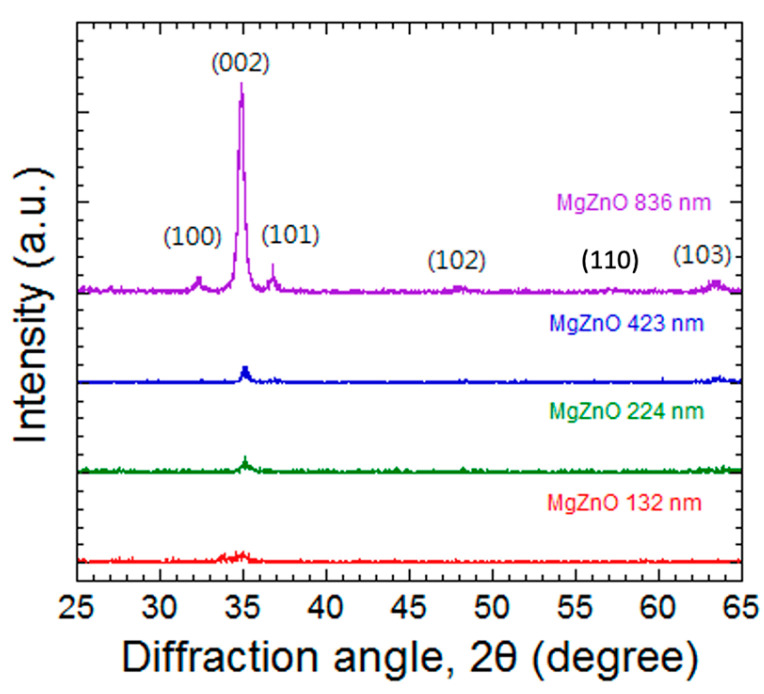
The XRD spectrum of the MgZnO films with different thicknesses.

The structural analysis of the MgZnO films was observed through an X-ray diffraction analysis as shown in Figure 1. The XRD results indicate that the MgZnO film has a polycrystalline structure, and it grows with a hexagonal wurtzite type. The significant peaks for the MgZnO film were ZnO (100), (002), (101), (102), (110), and (103) centered at 2θ, being 32.2°, 34.6° 36.4°, 48.0°, 57.0°, and 63.3°, respectively. Similar XRD results of ZnO film were reported by Wang et al. [26] and of Mg_0.1_Zn_0.9_O film [16]. The highest peak at 34.6° corresponds to the (002) plane of ZnO, indicating that the thin film layers are highly c-axis-oriented. It shows a slightly right shift as compared with the pure ZnO (002) peak, which is due to some of the Zn atoms being replaced by the Mg atoms and resulting in a smaller lattice constant. The XRD spectrum also shows that the crystallization of the film is improved as the thickness increases, for there is sufficient energy to drive the atoms and make a better crystallization.

**Figure 2:**
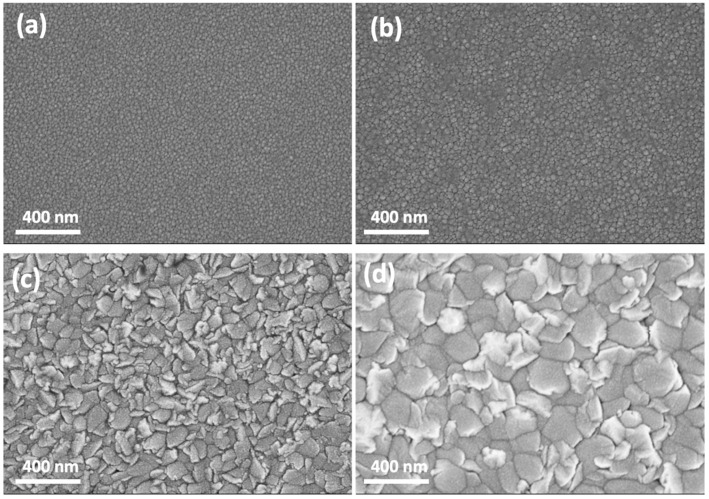
The SEM pictures of the surface morphology of the MgZnO film with the thickness of (**a**) 132 nm, (**b**) 224 nm, (**c**) 423 nm, and (**d**) 836 nm. The magnification of the SEM pictures is 50,000 times.

The surface morphologies of the films were observed through FE-SEM. The results are shown in Figure 2a–d. The grain size was evaluated by the scale bar of the SEM images. Twenty grains of each SEM image have been considered for the analysis. The evaluated size of each was given by a grain size range. For the film thicknesses of 132 and 224 nm, both show a smaller grain size. The grain size is around 20–30 nm for the 132 nm thickness MgZnO film and is around 35–45 nm for the 224 nm thickness MgZnO film. Both the films show smooth surface morphology. As the thickness of the MgZnO film increased, the average grain sizes increased also. The grain size is around 80–120 nm for the 423 nm thickness MgZnO film and is around 150–220 nm for the 836 nm thickness MgZnO film. The increase in the grain size consists of the growth mechanism of the thin film.

MgZnO is an n-type semiconducting metal oxide, where electrons make up the majority of the charge carriers. When MgZnO is exposed to air, the oxygen in the air interacts with the surface of the metal oxide and diffuses through the grain boundaries, which in turn gets absorbed by said boundaries. Then, the absorbed oxygen captures the electrons from the band of MgZnO and creates ionic oxygen species ($O_{2}^{-}$, $O^{-}$, $O^{2-}$) and which is dependent on the temperature and the following reactions take place at the interface [17,22]:

(1) $$O_{2}\to O_{2(abs)}$$

(2) $$O_{2(abs)}+e^{-}\to O_{2\left(abs\right)}^{-}\;at\;100\;{}^{\circ}C$$

(3) $$O_{2(abs)}^{-}+e^{-}\to 2O_{\left(abs\right)}^{-}\;at\;150\sim 200\;{}^{\circ}C$$

(4) $$2O_{\left(abs\right)}^{-}+e^{-}\to O_{\left(abs\right)}^{2-}\;at\;300\;{}^{\circ}C$$

Thus, a space-charged region is formed on the surface of the metal oxide, which creates a potential barrier for electron conduction. This will increase the material’s electrical resistance. With the introduction of a reducing gas such as hydrogen (*H_2_*) on the surface of the material, the resultant hydrogen atoms react with *O^−^* to extract electrons and produce water vapor.

(5) $$H_{2}+O_{\left(abs\right)}^{-}\to H_{2}O_{\left(gas\right)}+e^{-}$$

This donation of electrons increases the concentration of free carriers in the metal oxide, leading to a decrease in resistance. The presence of oxygen vacancies within the metal oxide lattice can further enhance this process by readily accepting electrons from nearby $O^{-}$, potentially leading to a larger change in resistance for films with more vacancies. Conversely, oxidizing gasses can accept electrons from the metal oxide and oxygen absorbed, depleting free carriers and increasing resistance. This change in resistance, dependent on the specific interaction between the gas and surface $O^{-}$, forms the core principle of metal oxide gas sensing.

**Figure 3:**
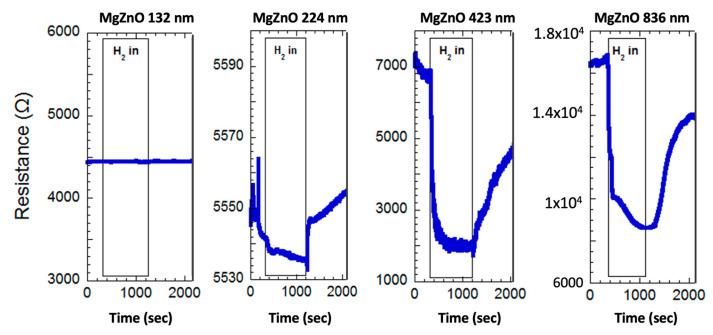
The variation of resistance of the MgZnO films concerning time during the hydrogen in/off period.

The MgZnO films with different film thicknesses were used as sensors to test the performance of the film toward hydrogen. The sensing characteristics of the MgZnO films with various thicknesses at the measurement temperature of 300 °C and the hydrogen concentration of 1000 ppm are shown in Figure 3. Their characteristic curves show the variation of resistance of the film with time.

The resistance fluctuation of the MgZnO sensor throughout the hydrogen in/out phase is depicted in Figure 3. Our investigation revealed thickness-dependent hydrogen sensing behavior in MgZnO films. Sensors with thicknesses of 132 nm and 224 nm exhibited minimal response (sensitivities of 1.00 and 1.02, respectively) under 1000 ppm hydrogen, suggesting their inadequacy for detection. Conversely, sensors with thicknesses of 423 nm and 836 nm displayed a noticeable decrease in resistance upon hydrogen exposure, with partial recovery upon removal (incomplete desorption). These sensors demonstrated improved sensitivity.

**Figure 4:**
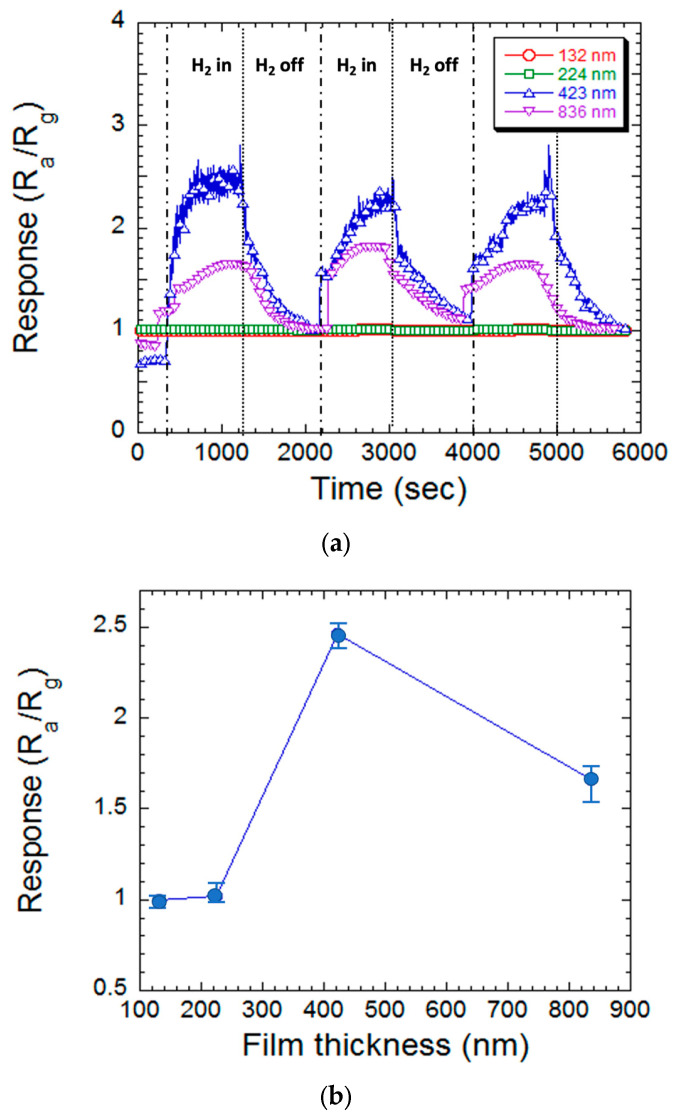
(**a**) The hydrogen sensing response of the MgZnO films with various thicknesses in a three-cycle period of hydrogen, on/off. (**b**) The hydrogen sensing response concerning the thickness of the MgZnO films.

**Figure 5:**
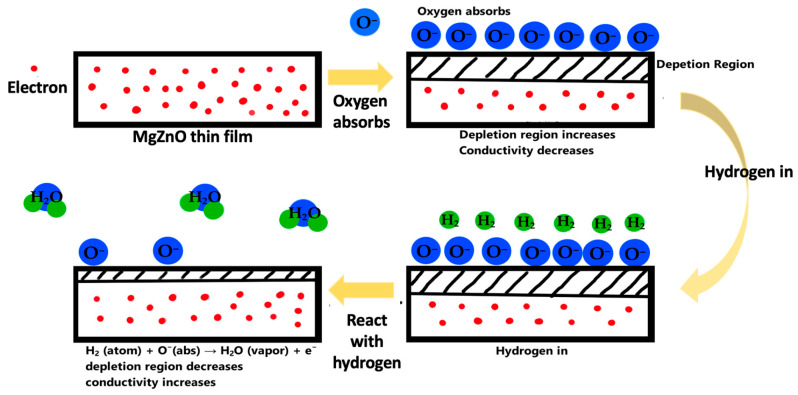
The sensing mechanism of the MgZnO films during the hydrogen in/off period.

Figure 4 and Figure 5 depict the comparative sensing response and thickness-dependent sensing response of MgZnO sensors with varying thicknesses (132, 224, 423, and 836 nm) exposed to 1000 ppm hydrogen cycles at 300 °C. The sensing response is defined as $Response,\;S=R_{a}/R_{g}$. The resistance measured in a gas environment was designated as *R_a_*, and that in an environment mixed with hydrogen concentration was designated as *R_g_*. The sensors exhibited a clear correlation between thickness and sensitivity (1.00, 1.02, 2.46, and 1.66, respectively). The samples of thicknesses of 132 and 224 nm show little response to hydrogen concentration. As the thickness of the MgZnO thin film increased to 423 nm, it shows a good sensing response of 2.46. Meanwhile, the sensing response degraded as the thickness increased to 836 nm and it is 1.66. Vijayalakshmi et al. [24] reported a sensing response of about 1.25 at the Mg:ZnO film, which was deposited at 1000 °C substrate heating and measured at room temperature of a 200 ppm hydrogen concentration. 

The response time and recovery time of the MgZnO film can be evaluated from Figure 4a. The response time is calculated as the time needed to reach 90% of the final state and the recovery time is evaluated as the time needed from the saturated value to 10% of the saturated value [27]. The 423 nm MgZnO thin film sensor has a response time of 280 s and recovery time of 450 s. Meanwhile, both response time and recovery time degraded to 450 s and 620 s, respectively, as the film thickness increased to 836 nm. Similar results had been reported by Vijayalakshmi et al. [24] and they showed a response time of 85 s and recovery time of 70 s at the Mg:ZnO nanorod sensor.

The observed trend suggests a positive correlation between film thickness and sensitivity. This can be attributed to the extended deposition time for thicker films during sputtering, which likely enhances defect formation. These defects, particularly oxygen vacancies, act as preferential absorption sites for oxygen, thus improving gas sensing performance [28]. However, the sensitivity reduction observed in the 836 nm sensor implies a trade-off. Larger grain sizes associated with increased thickness can decrease the total surface area available for gas interaction, thereby hindering sensitivity. These findings highlight the potential for improving sensor performance by tailoring the film’s morphology (the arrangement and structure of the material’s surface) to maximize the reactive surface area. This strategy could lead to the development of highly sensitive MgZnO-based hydrogen sensors.

The sensing mechanism of the MgZnO thin film is discussed as follows and shown in Figure 5. The variation in resistance is surface-controlled, which is based on the change in the conductivity of the material upon exposure to the H_2_ gas [29]. The surface of MgZnO thin films adsorb oxygen molecules and the adsorbed oxygen will become oxygen ions by capturing electrons from the conduction band of MgZnO semiconducting material [30]. This results in an increase in the depletion layer at the surface, which tends to increase resistance of the sensor before hydrogen sensing. As the MgZnO thin film is exposed in a H_2_ ambient atmosphere, the H_2_ molecule will react with oxygen ions, producing water vapor and releasing the trapped electrons back into the conduction band and decreasing the surface depletion layer. This will cause a decrease in the resistance of the film. The thicker MgZnO thin film shows more effective hydrogen sensing. This is due to rougher surface morphology and more defect formation. 

### 3.2. Temperature and Hydrogen Concentration Effect of MgZnO Film

**Figure 6:**
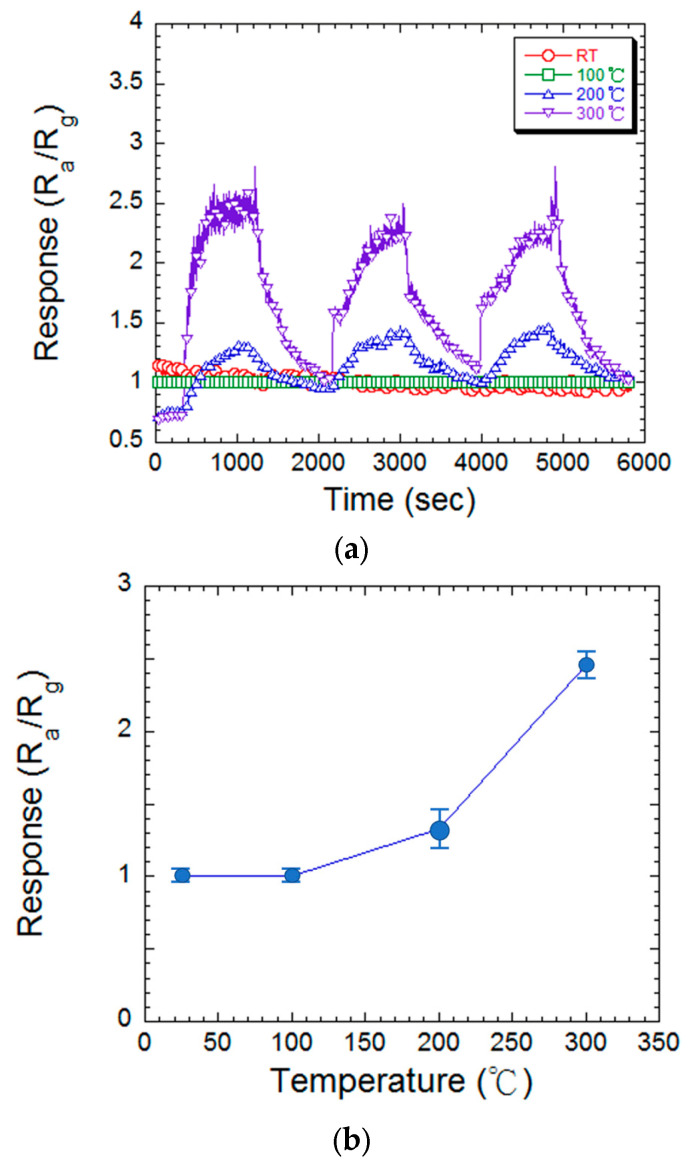
(**a**) The hydrogen sensing response of the MgZnO film at various measured temperatures. (**b**) The plot of sensing response concerning measured temperature.

This study investigated the hydrogen gas sensing characteristics of MgZnO thin films (thickness: 423 nm, Mg doping: 1.12 wt%) under various environmental conditions. As optimal performance in metal oxide semiconductor gas sensors relies on a specific operating temperature, the sensors were exposed to repeated hydrogen intake and release cycles at different temperatures. The corresponding changes in resistance were measured. Figure 6a,b depict the comparative sensitivity variations and the relationship between working temperature and sensing response.

The response of the MgZnO thin film sensor to hydrogen gas shows a substantial influence on the operating temperature. Data from experiments support this pattern. At room temperature and 100 °C, the sensor has a sensitivity of 1.00, suggesting inadequate hydrogen detection under these circumstances. The constraints of thermal energy at low temperatures (≤100 °C) explain this. Both the crucial surface interactions between absorbed hydrogen atoms and surface oxygen ions,

(6) $$H_{2}+O_{\left(abs\right)}^{-}\to H_{2}O_{\left(g\right)}+e^{-}$$

are impeded by insufficient thermal energy. Sensor sensitivity is reduced because of this slow contact. Thermal energy supports the processes as the operational temperature rises. An increase in the concentration of accessible hydrogen atoms for the reaction results from more efficient dissociation. Furthermore, a temperature increase encourages the $O^{-}$ ion to lose one of its electrons, producing water vapor (*H_2_O*) and a free electron. A boost in sensing response (1.33 at 200 °C; 2.46 at 300 °C) is caused by greater interaction between *H_2_* and $O^{-}$, for there is more thermal energy, which then causes the atom to have more kinetic energy, which in turn will produce more reactions on the sensor. At 150–200 °C and at 300 °C, 2$O^{-}$ and $O^{2-}$ oxygen ions are produced, respectively, at those temperatures, and there are more electrons that will be released during this when *H*_2_ and $O^{2-}$ react together. This will create an increase in free carriers in the metal oxide, which will decrease the resistance and therefore increase the response rate as shown in Figure 6a.

Overall, the tests show that the sensor responds minimally at room temperature and low temperatures, but that its sensitivity increases at higher operating temperatures. This phenomenon can be explained by the vital role that thermal energy plays in helping the hydrogen gas on the film surface to absorb and desorb. Elevated thermal energy improves the overall performance by reducing the energy barrier between the gas and the film [31].

**Figure 7:**
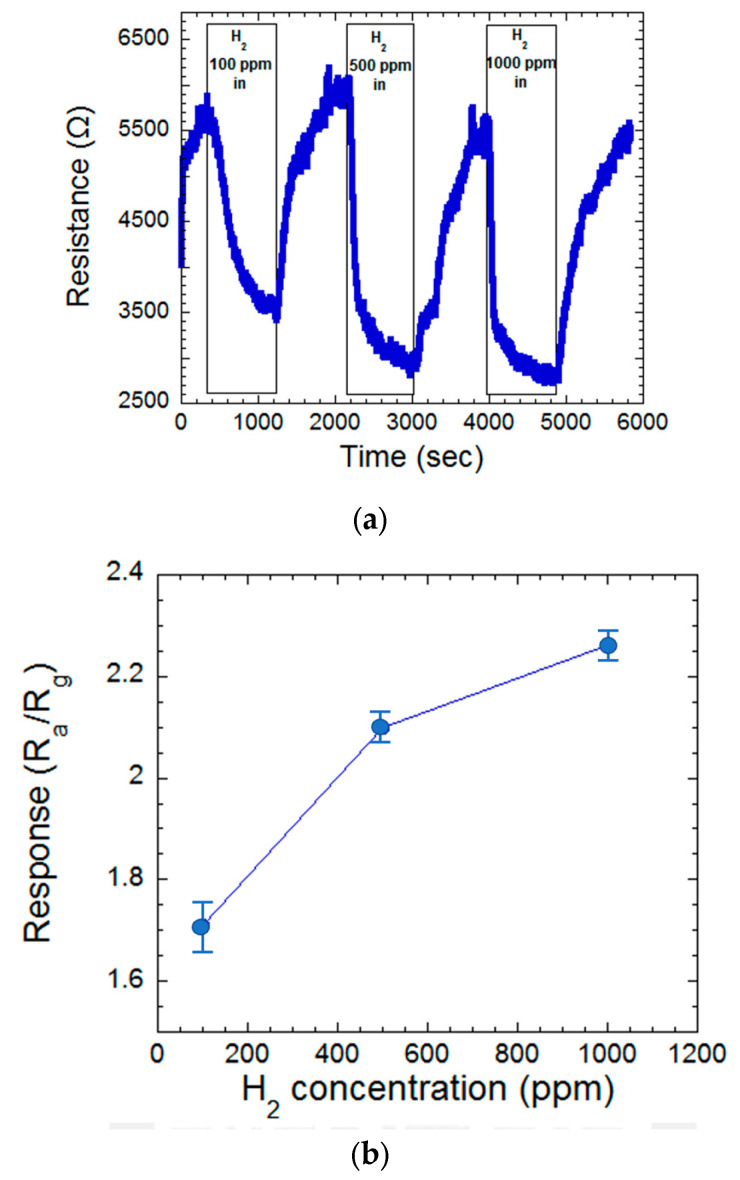
(**a**) The resistance variation of the MgZnO film at various hydrogen concentrations of in/off time. (**b**) The plot of sensing response concerning hydrogen concentration.

A key characteristic of effective gas sensors is a linear response to varying gas concentrations. Figure 7a,b depict the resistance changes observed in a MgZnO film at 300 °C when exposed to different hydrogen gas concentrations. The corresponding sensitivity values are plotted against the hydrogen concentration. The sensor exhibited a sensitivity of 1.71 at 100 ppm, increasing to 2.10 and 2.26 at 500 ppm and 1000 ppm hydrogen, respectively. These findings demonstrate a clear trend of increasing sensitivity with rising hydrogen concentration. This behavior confirms the sensor’s ability to effectively distinguish between different hydrogen gas concentrations within the tested range [22].

The 423 nm MgZnO thin film sensor shows a high sensing response of 2.64 at a hydrogen concentration of 1000 ppm at 300 °C measurement. Meanwhile, the sensing response decreased as the hydrogen concentration decreased. The sensing response is 1.71 at a 100 ppm hydrogen concentration at 300 °C measurement. Also, as the measured temperature decreased to room temperature, the sensing response decreased to zero. To develop a MgZnO thin film-based room temperature hydrogen sensor, the incorporation of UV light to enhance the sensing ability is a good choice [19,32]. The fabrication of a UV light/H_2_ gas dual sensing device is our future work.

## 4. Conclusions

A MgZnO thin film hydrogen sensor deposited by RF co-sputtering was studied in the research. The thickness effect of the MgZnO thin film sensor was stressed. It shows very low sensing response at the thinner thickness of the MgZnO film. Meanwhile, the sensing ability increases with thickness. It shows the best sensing response of 2.64 at the film thickness of MgZnO at a hydrogen concentration of 1000 ppm of 300 °C measurement. The high sensing abilities are both due to enhanced defect formation at the thick film. These defects, particularly oxygen vacancies, act as preferential absorption sites for oxygen, thus improving gas sensing performance.

## Data Availability

The original contributions presented in the study are included in the article, further inquiries can be directed to the corresponding author.
